# Improving Plant Growth and Alleviating Photosynthetic Inhibition and Oxidative Stress From Low-Light Stress With Exogenous GR24 in Tomato (*Solanum lycopersicum* L.) Seedlings

**DOI:** 10.3389/fpls.2019.00490

**Published:** 2019-04-16

**Authors:** Tao Lu, Hongjun Yu, Qiang Li, Lin Chai, Weijie Jiang

**Affiliations:** Key Laboratory of Horticultural Crops Genetic Improvement (Ministry of Agriculture), Institute of Vegetables and Flowers, Chinese Academy of Agricultural Sciences, Beijing, China

**Keywords:** photosynthesis, photoinhibition, endogenous GR24, tomato, low light

## Abstract

Low light (LL) is one of the main limiting factors that negatively affect tomato growth and yield. Techniques of chemical regulation are effective horticultural methods to improve stress resistance. Strigolactones (SLs), newly discovered phytohormones, are considered as important regulators of physiological responses. We investigated the effects of foliage spray of GR24, a synthesized SLs, on tomato seedlings grown under LL stress conditions. The results showed that application of GR24 effectively mitigated the inhibition of plant growth and increased the fresh and dry weight of tomato plants under LL. Additionally, GR24 also increased the chlorophyll content (Chl*a* and Chl*b*), the net photosynthetic rate (Pn), the photochemical efficiency of photosystem (PS) II (Fv/Fm), and the effective quantum yield of PSII and I [Y(II) and Y(I)], but decreased the excitation pressure of PSII (1-qP), the non-regulatory quantum yield of energy dissipation [Y(NO)] and the donor side limitation of PSI [Y(ND)] under LL. Moreover, application of GR24 to LL-stressed tomato leaves increased the electron transport rate of PSII and PSI [ETR(II) and ETR(I)], the ratio of the quantum yield of cyclic electron flow (CEF) to Y(II) [Y(CEF)/Y(II)], the oxidized plastoquinone (PQ) pool size and the non-photochemical quenching. Besides, GR24 application increased the activity and gene expression of antioxidant enzymes, but it reduced malonaldehyde (MDA) and hydrogen peroxide (H_2_O_2_) content in LL-stressed plants. These results suggest that exogenous application of GR24 enhances plant tolerance to LL by promoting plant utilization of light energy to alleviate the photosystem injuries induced by excess light energy and ROS, and enhancing photosynthesis efficiency to improve plant growth.

## Introduction

Strigolactones (SLs) are a general term for some natural witchweed alcohol compounds and their synthetic analogs. These compounds were isolated from the rhizosphere of cotton, a non-host plant of *Striga*, by Cook et al. (1972). However, they were identified in 2008 as a new class of plant hormones modulating development (Ruyter-Spira et al., 2011). Recent studies suggested that, besides the roles in stimulating seed germination, modulating root architecture, inhibiting shoot branching, modifying plant architecture, and promoting leaf senescence (Gomez-Roldan et al., 2008; Kapulnik et al., 2011; Ruyterspira et al., 2013; Hu et al., 2014), SLs are also positive regulators in plant responses to some abiotic stresses. Ha et al. (2014) found that *Arabidopsis thaliana* SL-response *max2* mutant exhibited hypersensitivity to drought and salt stress. Exogenous GR24, a synthetic SL analog, can enhance osmotic stress tolerance in *Lotus japonicus* and rescue the drought-sensitive phenotype of SL-deficient mutants like *max3* and *max4*, revealing multiple hormone-response pathways controlling the adaptation to environmental stress (Ha et al., 2014; Liu et al., 2015). Furthermore, pretreatment with GR24 could alleviate the salinity stress in rapeseed (*Brassica napus* L.) and dark chilling stress in pea and in *Arabidopsis* (Ma et al., 2017; Cooper et al., 2018). However, there is little information to elucidate the effects of SLs treatment on the physiological characteristic, photosynthesis and reactive oxygen metabolism in plants under low light stress.

Tomato (*Solanum lycopersicum* L.), a photophilous vegetable crop, is widely cultivated in greenhouse in northern China. Low light (LL) is one of the main environmental factors limiting tomato plant growth and crop productivity during winter and spring seasons. And, LL stress in tomato leads to numerous metabolic changes by inhibiting photosynthetic characteristics and disordering assimilation metabolisms (Barber and Andersson, 1992; Wang et al., 2010). Plants exposed to LL have thinner leaves and bad performance compared with the plants grown under normal growth conditions. Internal to leaves, LL stress causes limitation of photosynthesis by inhibiting photosynthetic efficiency (Murchie et al., 2005). Additionally, plants grown in LL have lower levels of photosystem (PS) II, ATP synthase, cytochrome (Cyt) *b/f*, and ribulose-1,5-bisphosphate carboxylase/oxygenase (Rubisco), as well as lower electron transport (ETR) and CO_2_ consumption (Leong and Anderson, 1984; Zivcak et al., 2014). And, LL stress often causes oxidative damage, which is manifested in the generation of reactive oxygen species (ROS). Then, several antioxidant enzymes, such as superoxide dismutase (SOD), peroxidase (POD), and catalase (CAT) are produced in stressed plants to scavenge ROS (Xu et al., 2010; Suzuki et al., 2012). Moreover, plants acclimated to LL showed lower biomass production and higher membrane lipid peroxidation (Hoglind et al., 2011). Under LL stress condition, crop plants with stronger resistance can enhance the tolerance to stress by improving the expression of specific genes and proteins. For examples, spermidine synthase genes were overexpressed in sweet potato and that improved the tolerance to LL stress (Ahmad et al., 2012). The increasing expression of ATP synthase β subunit protein enhanced the tomato resistance to LL stress (Cui et al., 2016). While, the overexpression of Rubisco activase gene may promote the low temperature and low light tolerance of cucumber plants (Bi et al., 2017).

In northern China, protected vegetable cultivation is frequently exposed to low light intensity owing to fog or haze. In an effort to devise new strategies for protection against such stresses, techniques of chemical regulation are commonly used and effective horticultural methods. In the present study, we investigated the effects of the function of exogenously-applied SLs on tomato seedlings under LL stress, hoping to alleviate the adverse effects of LL stress using a simple and environment friendly method, and to provide a new theoretical approach for improving the quality of tomato cultivation.

## Materials and Methods

### Plant Material and Experimental Conditions

Tomato seeds (cv. M82) were germinated at Chinese Academy of Agricultural Sciences in a semiautomatic glasshouse (average day/night temperatures of 25°C/15°C) and transferred to nutrition pots (13 cm × 13 cm) at the two-leaf stage under natural light (approximately 600 μmol m^-2^ s^-1^) at a relative humidity of 60%.

Seedlings reaching the six-leaf stage were transferred to a phytotron (plant growth sodium lamps as light source, 25°C/15°C) for 5 days to adapt the environment. Then they were divided into three phytotrons with 30 pots in each part. Two groups (LL+H_2_O and LL+GR24) were subjected to LL treatment under photosynthetic photon flux density (PPFD) of 170 ± 30 μmol m^-2^ s^-1^ and were sprayed with H_2_O and with 15 μM GR24, respectively. The control group was sprayed with H_2_O and kept under a PPFD of 450 ± 30 μmol m^-2^ s^-1^. Each group was sprayed with the appropriate solution once a day for 7 days (see Supplementary Figure S1). Environmental conditions were as follows: photoperiod of 12 h light/12 h dark, temperature of 25°C/15°C, and relative humidity of 60%. All groups were instituted at day 0 and irrigated with half-Hoagland’s nutrient solution simultaneously. Measurements were performed on the fourth fully expanded tomato leaf with four replicates.

### Measurement of Morphological Index, Photosynthetic Pigment Content, and Biomass Distribution

Plant height and stem diameter were measured by ruler and electronic Vernier caliper, respectively. Chlorophyll contents were determined from 0.5 g fresh leaf samples, which were extracted in 80% acetone for 24 h in darkness. Absorbance value was measured at 645 and 663 nm. The concentration of chlorophyll pigments were calculated by the following equations (Mackinney, 1941; Porra, 2002): Chl*a* (mg ⋅ g^-1^) = (12.72A_663_–2.59A_645_) × V/(1000 × W); Chl*b* (mg ⋅ g^-1^) = (22.88A_645_–4.67A_663_) × V/(1000 × W).

### Measurement of Chlorophyll Fluorescence, P700 and Gas-Exchange Parameters

Chlorophyll fluorescence and P700 parameters were measured by Dual-PAM-100/F fluorometer (Heinz Walz Gmbh, Effeltrich, Germany). Seedlings were dark-adapted for 20 min. First, the leaf surface was exposed to the modulated measuring light (0.6 kHz, PPFD ≤ 0.1 μmol m^-2^ s^-1^, “weak red light”) to measure initial fluorescence (Fo). Then, the saturation pulse light (20 kHz, 300 ms pulse of 10000 μmol m^-2^ s^-1^, “white light”) was applied to determine maximal fluorescence (Fm) and maximal change in P700. Next, the actinic light (AL, 531 μmol m^-2^ s^-1^) was used to stimulate normal photosynthesis for several minutes. During illumination, steady-state fluorescence (Fs) and maximal fluorescence in this light (Fm′) were obtained. Finally, initial fluorescence in this light (Fo′) was acquired when AL was turned off and the far-red light (FR) was switched on. The maximum photochemical efficiency of PSII (Fv/Fm), efficiency of excitation energy capture by open PSII reaction centers (Fv′/Fm′), PSII excitation pressure (1-qP) and non-photochemical quenching coefficient (NPQ) were calculated as follows: Fv/Fm = (Fm-Fo)/Fm; Fv′/Fm′ = (Fm′-Fo′)/Fm′; 1-qP = 1-(Fm′-Fs)/(Fm′-Fo′); NPQ = Fm/Fm′-1 (Florian et al., 2009). Additionally, the quantum efficiency of PSII photochemistry [Y(II)] and quantum yields of non-regulated and regulated energy of PSII [Y(NO) and Y(NPQ)] were calculated as follows: Y(II) = (Fm′-Fs)/Fm′; Y(NO) = Fs/Fm; Y(NPQ) = 1-Y(II)-Y(NO) (Genty et al., 1989; Busch et al., 2009; Yamori et al., 2011).

The effective quantum yield of PS I [Y(I)] was also measured using the saturation pulse method and determined from the acceptor-side and donor-side limitations of PSI [Y(NA) and Y(ND)], which were calculated as follows: Y(NA) = (Pm-Pm′)/Pm; Y(ND) = 1-P700red; Y(I) = 1-Y(NA)-Y(ND). The maximum oxidation state of PSI (Pm) was recorded by applying a saturation pulse in the presence of FR, and Pm′ was measured similarly to Pm without FR pre-illumination (Klughammer and Schreiber, 1994; Lu et al., 2017b).

Electron transport rate was calculated by ETR = (Quantum photosynthetic yield) × PAR × 0.84 × 0.5. Notably, if cyclic electron flow (CEF) was activated, ETR(II) was smaller than ETR(I). Although CEF might have been overestimated, we considered the trend of change in the ratio of CEF to linear electron flow (LEF) to be reliable. Hence, we used the parameters Y(CEF) = Y(II)-Y(I) and Y(CEF)/Y(II) = [Y(I)-Y(II)]/Y(II) to estimate the extent of CEF (Miyake et al., 2005; Huang et al., 2010; Zhang et al., 2014).

Gas-exchange parameters were determined by a portable photosynthesis system (Li-6400XT, Li-COR, Inc., United States). Each leaf was balanced for 1 min in the leaf chamber with constant irradiation (600 μmol m^-2^ s^-1^). Leaf temperature and CO_2_ concentration were maintained at 25°C and 400 ppm, respectively.

### Measurement of rETR Light Response Curve, “OJIP” Curve, JIP-Test, and PQ Pool

The rapid light curves of relative electron transport rate (rETR) were measured based on Ralph and Gademann (2005): the light intensity gradient of PAR was set as 0, 59, 79, 113, 156, 225, 320, 457, 648, 958, and 1407 μmol m^-2^ s^-1^ for 20 s each. The data were entered into the Origin software with a fitting model equation: rETR = rETR_max_ ⋅ E_k_ ⋅ (α ⋅ PAR/ rETR_max_), where α is the initial slope of the line at low PAR, ETR_max_ is the maximum electron transport rate and E_k_ is the minimum saturation level.

The fast acquisition kinetics of Chl a fluorescence was monitored as described by Schreiber et al. (1995). Leaves were kept in darkness for 20 min and then received strong continuous illumination to measure the rapid acquisition kinetics. The relative variable fluorescence (Vt) is the ratio of variable fluorescence to maximal variable fluorescence, i.e., Vt = (Ft-Fo)/(Fm-Fo), where Ft is a given time fluorescence. To obtain the “OJIP” curves, a log time scale assessment of the relative variable fluorescence (Fluo, V) was performed to reflect the states of the PSII acceptor and donor sides (Lazar, 2006).

According to the JIP-test method (Zeliou et al., 2009), many parameters reflecting the structure and function of photosynthetic apparatus were calculated: (i) specific fluxes or activities: average absorption per active reaction center (RC), ABS/RC = Mo ⋅ (1/Vj) ⋅ (1/φPo); flux of excitons trapped per active RC, TRo/RC = Mo ⋅ (1/Vj); electron transport per active RC, ETo/RC = Mo ⋅ (1/Vj) ⋅ φo; ratio of total dissipation to the amount of active RC, DIo/RC = ABS/RC-ETo/RC; (ii) quantum efficiencies or flux ratios: maximum yield of primary photochemistry, φPo = TRo/ABS; quantum yield of heat dissipation, φDo = Fo/Fm; quantum yield of absorbed photons for electron transport, φEo = ETo/ABS; quantum yield of reduction in final electron acceptors of PSI per photon absorbed, φRo = TRo/ABS(1-Vi); the efficiency to conserve trapped excitation energy as redox energy, φo = ETo/TRo; (iii) performance index based on the absorption of light energy, PI(abs) = (RC/ABS) ⋅ [φPo/(1-φPo)] ⋅ [φo/(1-φo)]. Here, Mo = 4 ⋅ (F300 μs-Fo)/(Fm-Fo), Vj = (F2 ms-Fo)/(Fm-Fo), and Vi = (F30 ms-Fo)/(Fm-Fo).

*In vivo*, plastoquinone pool (PQ) size was determined as follows: the FR light was activated at 0 s, then a single turnover flash (50 μs, PQ pool being oxidized) was used at 60 s, and the multiple turnover flash light (50 ms, PQ pool is fully reduced) was used at 100 s; when the curve tended to equilibrium at 160 s, the measurement was terminated. The complementary areas ST-area and MT-area were used to compute the functional PQ pool size, that is, e^-^/P700 = MT-area/ST-area (Savitch et al., 2001; Zhang et al., 2014).

### Activity of Antioxidant Enzymes, Malondialdehyde (MDA), and Hydrogen Peroxide (H_2_O_2_) Determinations

The activities of SOD, CAT, and POD were determined by specific enzyme activity detection kit provided by Nanjing Jiancheng Bioengineering Institute with separate 0.1 g samples. The concentrations of MDA and H_2_O_2_ were measured by MDA and H_2_O_2_ assay kit (Keming, Suzhou, China) with separate 0.2 g samples.

### Extraction of Total RNA and RT-PCR Analysis

Total RNA was extracted with an RNAprep Pure Plant Total RNA Extraction Kit (Tiangen Biotech, Co., Ltd., Beijing, China). Then, cDNA was acquired by reverse transcription of RNA samples with a cDNA Synthesis Kit (Yesen Biotech, Co., Ltd., Shanghai, China). The nucleic acid concentrations of total RNA and cDNA were determined from the A_260_ and A_280_ values by a NanoDrop 2000c (Thermo Fisher Scientific, Inc., Waltham, MA, United States). The primer sequences are listed in Supplementary Table S1. Each cDNA sample was used as a template and mixed with primers and qPCR SYBR^®^ Green Master Mix (Yesen Biotech, Co., Ltd.). RT-PCR analysis was performed by iQ5 (Bio-Rad Laboratories, Inc., United States). To obtain relative gene expression for each sample, the threshold cycle (C_t_) value was normalized to *actin* and compared to the control samples according to the 2^-ΔΔCt^ method. Each sample was evaluated with three replications.

## Results

### Key Growth Parameters

Low light stress was reflected in not only the morphological indices, but also the biomass allocation of tomato plants (Table 1 and Supplementary Figure S2). The stem diameter of the LL+H_2_O group was significantly decreased by 13%, while plant height was significantly increased by 38%, compared to those of non- stressed tomato plants. Application of GR24 under LL effectively improved those indices by 9 and 8%, respectively. The contents of Chl*a* and Chl*b* under LL stress were significantly lower than those of the control group. However, exogenous GR24 significantly increased those photosynthetic pigment contents by 42 and 61%, respectively, compared to those of the LL+H_2_O group. LL stress also negatively affected both total fresh weight (FW) and total dry weight (DW) of tomato plants as they were significantly reduced by 20 and 29% respectively compared to the control group, whereas, with the application of GR24 under LL stress, there were significant increases of 6 and 15%, respectively.

**Table 1:** Effects of exogenous GR24 on morphogenesis, photosynthetic pigment content, and fresh and dry weights of tomato plants grown under LL stress.

Treatments	Plant height (cm)	Stem diameter (mm)	Chl(*a*) (mg ⋅ g^-1^)	Chl(*b*) (mg ⋅ g^-1^)	Chl(*a/b*)	Total FW (g)	Shoot FW (g)	Root FW (g)	Total DW (g)	Shoot DW (g)	Root DW (g)
Control	24.99 ± 1.88^c^	6.93 ± 0.38^a^	2.27 ± 0.29^a^	1.43 ± 0.10^a^	1.64 ± 0.31^a^	17.84 ± 0.67^a^	13.95 ± 0.48^a^	3.86 ± 0.28^a^	1.57 ± 0.08^a^	1.25 ± 0.06^a^	0.32 ± 0.03^a^
LL+H_2_O	37.44 ± 1.75^a^	6.05 ± 0.32^b^	1.39 ± 0.10^b^	0.61 ± 0.05^c^	2.28 ± 0.33^a^	14.25 ± 0.31^c^	11.99 ± 0.50^b^	2.25 ± 0.14^b^	1.11 ± 0.07^c^	0.89 ± 0.08^c^	0.23 ± 0.03^b^
LL+GR24	34.11 ± 1.21^b^	6.52 ± 0.30^ab^	1.98 ± 0.16^a^	0.98 ± 0.15^b^	2.08 ± 0.43^a^	15.08 ± 0.46^b^	12.42 ± 0.39^b^	2.67 ± 0.30^b^	1.30 ± 0.09^b^	1.01 ± 0.08^b^	0.29 ± 0.03^a^

*Data are the means of four replicates with standard errors (Values are significantly different at *P* ≤ 0.05 in the same column by different letters, based on the LSD test)*.

### Photosynthesis and PSII Function

**Figure 1:**
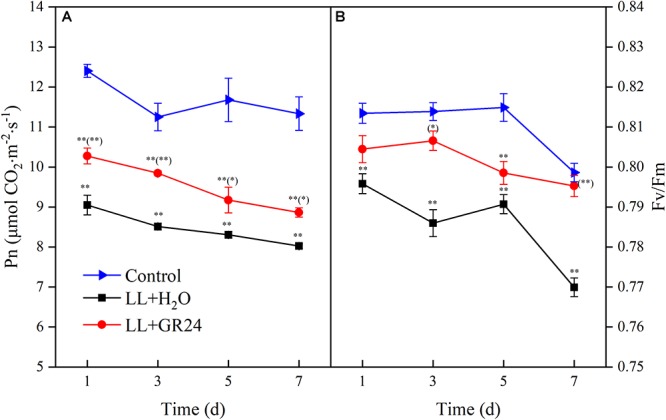
Effects of exogenous GR24 on the net photosynthetic rate **(A)** and PSII activity **(B)** of tomato leaves under LL stress. Data are the means of four replicates with standard errors shown by vertical bars. ^∗^ and ^∗∗^ indicate statistically significant differences at *P* ≤ 0.05 and *P* ≤ 0.01, respectively. Multiple comparisons between the figures among the treatments under LL are shown in parentheses.

In order to compare the plant photosynthetic performance of different treatments, same acting light intensity (nearby light saturation point of control group) was used. Compared with control, the net photosynthetic rate (Pn) and the maximal photochemical efficiency of PSII (Fv/Fm) decreased significantly under LL stress. In addition, the degree of inhibition degree was aggravated with prolonged treatment time (Figure 1). However, GR24 application led to a marked improvement of Pn under LL, although the Pn value was still significantly lower than that of the control throughout the treatment process (Figure 1A). Additionally, Fv/Fm increased significantly with application of GR24, nearly reaching the control level on days 3 and 7 (Figure 1B).

**Figure 2:**
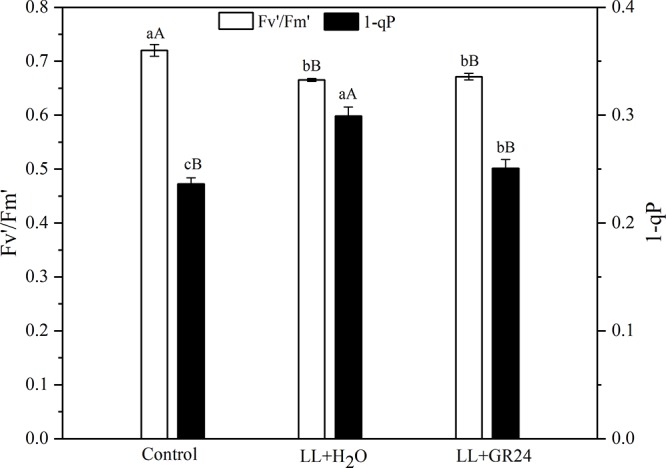
Effects of exogenous GR24 on Fv′/Fm′ and 1-qP of tomato leaves under LL stress on the 7^th^ day of treatment. Fv′/Fm′, efficiency of excitation capture by open PSII centers; 1-qP, excitation pressure of PSII. Lowercase and capital letters represent significant differences between treatments at *P* ≤ 0.05 and *P* ≤ 0.01 by the LSD test, respectively.

The capture efficiency by open PSII centers (Fv′/Fm′) of the LL+H_2_O group was significantly decreased by 7%, while the excitation pressure of PSII (1-qP) was significantly increased by 25% compared to non- stressed tomato plants (Figure 2). No significant effect of GR24 application on Fv′/Fm′ was obtained; however, 1-qP was significantly decreased by 17% compared with the LL+H_2_O group. Compared to the control group, the maximal and initial fluorescence (Fm and Fo) significantly decreased by 8 and 13% respectively, while the NPQ significantly increased by 24% (Table 2) under LL stress. In addition, exogenous GR24 effectively increased Fm, Fo and NPQ by 3, 10, and 7% respectively, as compared to LL+H_2_O stressed tomato plants.

**Table 2:** Effects of exogenous GR24 on dark-adapted maximum and initial fluorescence (Fm and Fo) and non-photochemical quenching coefficient (NPQ) of tomato seedlings under LL stress on the 7^th^ day of treatment.

Treatments	Fm	Fo	NPQ
Control	1.16 ± 0.03^aA^	0.24 ± 0.01^aA^	0.69 ± 0.07^cB^
LL+H_2_O	1.07 ± 0.03^bA^	0.21 ± 0.01^bB^	0.86 ± 0.05^bA^
LL+GR24	1.11 ± 0.04^aA^	0.23 ± 0.01^aA^	0.92 ± 0.04^aA^

*Lowercase and capital letters represent significant differences difference between treatments at *P* ≤ 0.05 and *P* ≤ 0.01 by the LSD test, respectively*.

### Light Energy Distribution and Electron Transport Between the Photosystems

**Figure 3:**
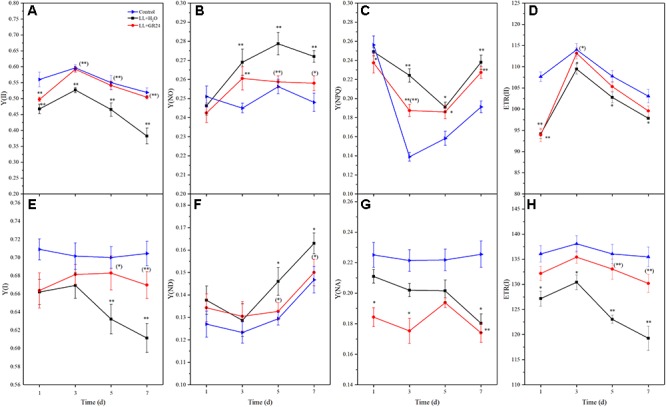
Effects of exogenous GR24 on intersystem energy distribution and electron transport rate (ETR) of tomato seedlings under LL stress. Y(II), effective quantum yield of PSII **(A)**; Y(NO), fraction of energy that is passively dissipated in form of heat and fluorescence **(B)**; Y(NPQ), fraction of energy dissipated in form of heat via the regulated non-photochemical quenching mechanism **(C)**; ETR(II), ETR of PSII **(D)**; Y(I), effective quantum yield of PSI **(E)**; Y(ND), fraction of overall P700 that is oxidized in a given state **(F)**; Y(NA), fraction of overall P700 that cannot be oxidized in a given state **(G)**; ETR(I), ETR of PS I **(H)**. ^∗^ and ^∗∗^ indicate statistically significant differences at *P* ≤ 0.05 and *P* ≤ 0.01, respectively. Multiple comparisons between the figures among the treatments under LL are shown in parentheses.

In this study, we measured direct information regarding both PSII and PSI *in vivo*. LL stress inhibited the effective quantum yield of PSII [Y(II)], which was significantly lower than control. However, Y(II) was significantly promoted by application of GR24 compared to the LL+H_2_O group, and it recovered to a similar level to that of control after 3 days (Figure 3A). Compared to the control, the regulatory and non-regulatory quantum yields of energy dissipation [Y(NPQ) and Y(NO)] were both significantly increased by LL stress. Once GR24 was applied, Y(NO) was decreased significantly compared with that of LL+H_2_O. However, Y(NPQ) showed no differences between these two treatments after 5 days (Figure 3B,C). The quantum yield of PSI photochemistry [Y(I)] decreased gradually with prolonged LL stress due to a decline in the acceptor-side limitation of PSI [Y(NA)] and an increase in the donor-side limitation of PSI [Y(ND)]. By applying GR24, Y(I) and Y(ND) could be restored to the control levels and showed significant difference from the LL+H_2_O group on the 7^th^ day (Figure 3E–G).

Additionally, the electron transport rates of both photosystems [ETR(II) and ETR(I)] were significantly reduced by LL stress (Figure 3D,H). Application of GR24 slightly promoted ETR(II) and significantly increased ETR(I) in tomato leaves under LL stress. LL stress inhibited linear electron transfer while stimulating the CEF around PS I [Y(CEF)], which was increased by 12%. GR24 treatment further stimulated CEF, but there were no significant differences among the three groups (Supplementary Figure S3a). Overall, exogenous GR24 significantly increased the ratio of the quantum yield of CEF to Y(II) [Y(CEF)/Y(II)] by approximately 90 and 74% compared to the LL+H_2_O and control groups, respectively (Supplementary Figure S3b).

### Expression of PSII and PSI Reaction Center Genes

**Figure 4:**
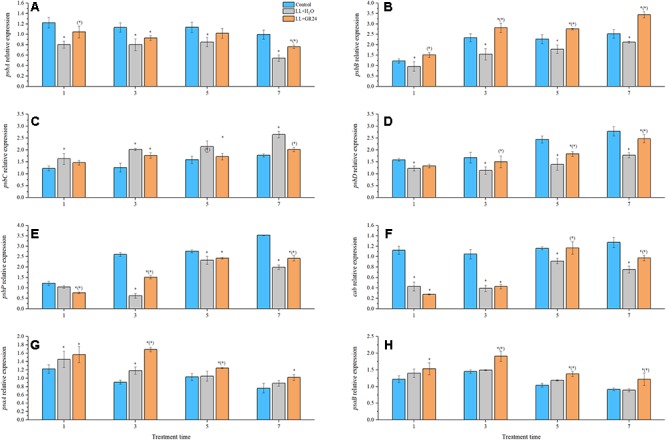
Effects of exogenous GR24 on the expression of genes encoding the PSII and PSI reaction center core proteins of tomato seedlings under LL stress. Expression levels of the corresponding genes at day 0 were used as control. RT-PCR was conducted with three replications. ^∗^ Indicate statistically significant differences at *P* ≤ 0.05. Multiple comparisons between the figures among the treatments under LL are shown in parentheses. **(A)** The expression of *psbA*; **(B)** the expression of *psbB*; **(C)** the expression of *psbC*; **(D)** the expression of *psbD*; **(E)** the expression of *psbP*; **(F)** the expression of *cab*; **(G)** the expression of *psaA*; **(H)** the expression of *psaB*.

Compared with control, the expression of genes encoding the PSII reaction center (e.g., *psbA*, *psaB*, *psbD*, *psbP*, and *cab*) was significantly decreased by LL stress during the entire experimental period. Additionally, application of GR24 considerably increased the expression of *psbA*, *psbB*, *psbD*, *psbP*, and *cab* at the 7^th^ day by 40, 48, 78, 11, and 5% respectively, compared to that of LL+H_2_O plants (Figure 4A,B,D–F). Interestingly, the relative expression of *psbC* tended to increase gradually with prolonged treatment time. At the same time, exogenous GR24 significantly decreased that tendency compared with that in LL+H_2_O, and no significant difference was obtained between the GR24 treatment and the control (Figure 4C). The PSI reaction center consists of the A1 and A2 proteins encoded by *psaA* and *psaB*, respectively. Exogenous GR24 significantly increased the expression of both these genes compared to that in LL+H_2_O (Figure 4G,H). However, no significant differences were found in the expression levels of these two genes between the LL+H_2_O and control groups during post-processing, although expression in the former was higher than that in the latter. In addition, application of GR24 significantly upregulated the expression of *psaB* from 3 days relative to that in the LL treatment, and significantly increased *psaA* expression at 3, and 5 days but not at 7 days.

### Performance of RLC, PIFT, and P700^+^ Re-reduction Curves

**Figure 5:**
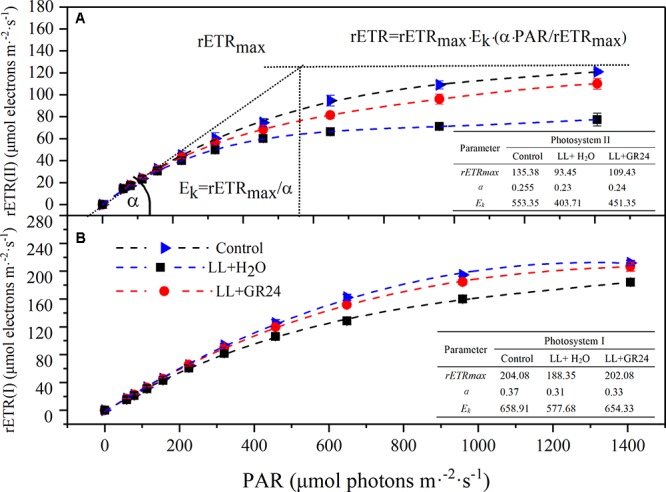
Effects of exogenous GR24 on rETR-PAR response curve and fitting parameters of tomato seedlings under LL stress on the 7^th^ day of treatment. Curves were fitted with OriginLab software, and the fitting parameters were determined by Dual-PAM measuring software, as shown in the embedded tables. The fitted curves are displayed as dotted lines. Fitted equation: *rETR* = *rETR_max_* ⋅ *E_k_* ⋅ *(α* ⋅ *PAR*/*rETR_max_)*; *rETR*, relative electron transport rate; *rETR_max_*, maximum potential relative electron transport rate; *α*, the initial slope of the rapid light curve; *E_k_*, semi-light saturation point. **(A)** The rETR(II) light response curve and fitting parameters, **(B)** the rETR(I) light response curve and fitting parameters.

Three average rapid light response curves (RLCs) of rETR ranging from 0 to 1500 μmol m^-2^ s^-1^ were used to investigate the photosynthetic capacity of the tomato plants. The rETR in tomato leaves rapidly increased with increasing light intensity. However, the rETR became steady after the light intensity reached 800 μmol m^-2^ s^-1^. The upper curve (control) showed the highest rETR, while rETR(II) and rETR(I) under LL were only 50 and 70% of the control when the light intensity reached 1400 μmol m^-2^ s^-1^. GR24 application effectively improved the rETR, as exhibited by the middle curve (Figure 5). The slopes of the light-limiting regions (α) of the three curves were similar. The H_2_O-treated plants under LL reached plateaus (*E_k_*) in rETR(II) and rETR(I) relatively quickly (404 and 578 μmol m^-2^ s^-1^, respectively), whereas the GR24-treated plants required approximately 450 and 650 μmol m^-2^ s^-1^; the values in both treatments were lower than those in the control. Application of GR24 also effectively increased the maximum potential relative electron transport rates (*rETR_max_*) of PSII and PSI by 17 and 7% respectively, compare to the LL+H_2_O group.

Post-illumination chlorophyll fluorescence transient (PIFT) has potential as a new tool for investigating how photosynthesis is regulated, the slope of the rising curve suggests the state of CEF (Supplementary Figure S4a). The P700^+^ re-reduction curves reflect dynamic changes in P700 oxidation state, and the slope of the falling curve indicates the rate of CEF (Supplementary Figure S4b). Our data showed that the plants in the G24 treatment had the greatest slope values in both PIFT and P700^+^ re-reduction curves, followed by the LL+H_2_O and the control groups.

### Analysis of O-I_1_-I_2_-P, JIP-Test, and PQ Size

**Figure 6:**
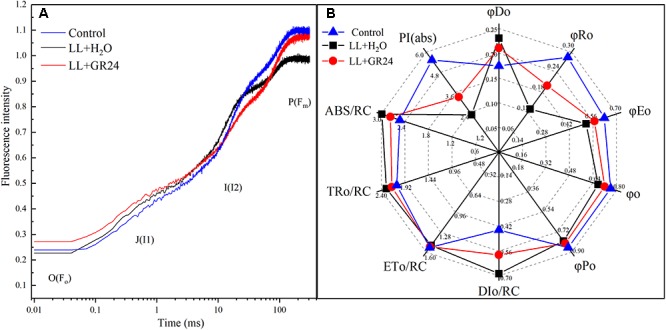
Effects of exogenous GR24 on the performance of rapid induction kinetics **(A)** and JIP-test analysis **(B)** of tomato seedlings under LL stress on the 7^th^ day of treatment. PI(abs), performance index based on the absorption of light energy; ABS/RC, average absorption per active RC; TRo/RC, flux of exciton trapped per active RC; ETo/RC, electron transport per active RC; DIo/RC, ratio of total dissipation to the amount of active RC; φPo, maximum yield of primary photochemistry; φo, the efficiency to conserve trapped excitation energy as redox energy; φEo, quantum yield of absorbed photons for electron transport; φRo, quantum yield of reduction of final electron acceptors of PSI per photon absorbed; φDo, quantum yield of heat dissipation.

Figure 6 shows the logarithmic time scale of the rapid induction kinetics (O-I_1_-I_2_-P). The level of Fo is considered a pronounced step. Under saturating light with a given intensity, the half-rise time of Fo-I_1_ (photochemical phase) is approximately 100 μs. The level of I_1_ is characterized by another pronounced step, and the ‘thermal’ I_1_-I_2_ and I_2_-F_m_ stages follow. The results showed that the relative variable fluorescence (v) of the Fo-I_1_ and I_1_-I_2_ phases of the LL+H_2_O group was higher than that of the control group, but GR24 application decreased that v. In contrast, LL greatly decreased v at the I_2_-F_m_ stage, while application of GR24 increased that v (Figure 6A). JIP-test analysis showed that LL stress decreased the values of φRo, φEo, φo, and φPo by 30, 9, 5, and 4%, respectively. In contrast, GR24 treatment increased those values by 35, 9, 6, and 3% compared with those of the LL+H_2_O group. Meanwhile, the values of DIo/RC, ABS/RC, and TRo/RC were increased by 32, 10, and 5% under LL stress conditions, while application of GR24 effectively mitigated them by 18, 8, and 5%, respectively. In addition, LL stress had a large negative effect on leaf performance index PI(abs), which was reduced by 40% compared to that of the plants under control condition. However, exogenous GR24 could significantly increase it by 32% compared with the LL+H_2_O group (Figure 6B).

Low light stress significantly decreased the PQ pool size in tomato leaves by 28% compared to that of non-stressed tomato seedlings. GR24 treatment significantly mitigated this effect by 16% compared with that in the LL+H_2_O group, although the oxidized PQ pool in GR24-treated plants was still significantly smaller than that in the controls (Supplementary Figure S5).

### ROS Metabolism and Lipid Peroxidation Assay

In the present study, LL treatment progressively increased the MDA and H_2_O_2_ contents of tomato seedlings by 1.2 and 0.5 times, respectively (Table 3). The difference between the LL treatment and control was highly significant, application of GR24 significantly reduced the H_2_O_2_ content to the control level, 26% lower than the LL+H_2_O group. In addition, MDA content was significantly reduced by 33% in GR24-treated tomato leaves.

**Table 3:** Effects of exogenous GR24 on MDA and H_2_O_2_ contents of tomato leaves under LL stress.

Treatment	MDA content	H_2_O_2_ content
7 days	nmol ⋅ g^-1^FW	μmol ⋅ g^-1^FW

Control	21.45 ± 5.39^cB^	1.58 ± 0.16^bB^
LL+H_2_O	48.06 ± 8.28^aA^	2.45 ± 0.21^aA^
LL+GR24	32.09 ± 2.43^bB^	1.81 ± 0.04^bB^

*Lowercase and capital letters represent significant differences difference between treatments at *P* ≤ 0.05 and *P* ≤ 0.01 by the LSD test, respectively*.

**Figure 7:**
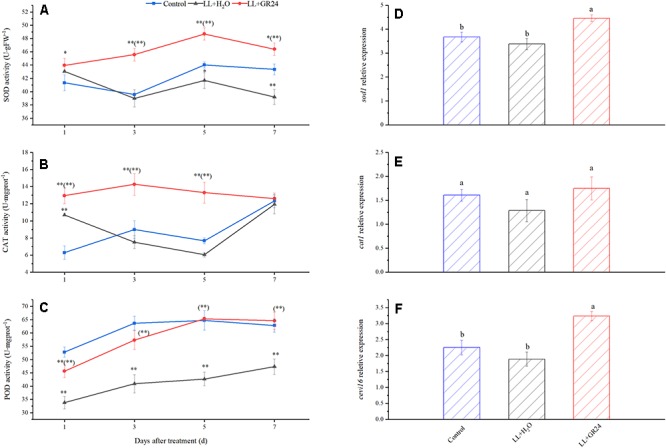
Effects of exogenous GR24 on the activity of SOD **(A)**, CAT **(B)**, POD **(C)**, and the transcript level of sod1 **(D)**, cat1 **(E)**, and cevi16 **(F)** in tomato leaves under LL stress. The transcript level shown in the figure are from the 7^th^ day of treatment. ^∗^ and ^∗∗^ indicate statistically significant differences at *P* ≤ 0.05 and *P* ≤ 0.01, respectively. Multiple comparisons between the figures among the treatments under LL are shown in parentheses. Lowercase letters represent significant differences difference between treatments at *P* ≤ 0.05 by the LSD test.

Furthermore, LL increased SOD activity but decreased POD activity significantly, while CAT activity was not significantly affected. Nevertheless, GR24 application significantly increased the activities of the above three enzymes compared with those in the LL+H_2_O group, and, POD activity was maintained at the control level (Figure 7A–C). In addition, exogenous GR24 under LL significantly promoted the gene expression of *sod1* and *cevi16* by 32 and 72%, respectively. Although application of GR24 increased *cat1* gene expression by 9 and 36% compared with the control and LL+H_2_O groups, respectively, no significant differences were obtained among the three treatments (Figure 7D–F).

## Discussion

In general, light directly effects plant’s photosynthesis and photomorphogenesis. The former supplies necessary energy for the formation of chlorophyll and biomass; the latter controls plant growth and development by light signals. The change of plant biomass is a comprehensive embodiment of its response to adverse stresses (Gaba and Black, 1983; Shu et al., 2016). In view of the results presented herein, it is clear that tomato plants showed disturbances of growth and development such as inhibited plant growth elongated petioles, decreased biomass production and so on after LL treatment, while GR24 treatment relieved the symptoms of LL stress (Table 1 and Supplementary Figure S2), indicating that GR24 application could maintain a better photosynthetic and nutrient absorption metabolic level of tomato plants under LL (Barber and Andersson, 1992; Xu et al., 2010; Yuan et al., 2010). The role of SLs in branching is further supported by studies done in pea and rice *ccd8* mutants, *rms1* and *d10*, respectively. Both mutants are deficient in SLs and the branching phenotype is rescued by treatment with GR24 and natural SLs (Arite et al., 2007; Gomez-Roldan et al., 2008). All these studies established SLs as a negative regulator of branching.

The cell organelle like chloroplast, which is the site for most of photosynthetic processes is also affected by LL stress (Xu et al., 2010; Zhou et al., 2015). The chlorophyll (Chl) content is used as an indicator of chloroplast development and photosynthetic performance. In this work, the observed increases of Chl*a* and Chl*b* content under LL by GR24 application (Table 1) might refer to the inhibition of Chl-degrading enzyme activity by relating chlorophyllase, which is consistent with reports by Naeem et al. (2012). Moreover, GR24 could regulate the combination of Chl and membrane proteins, to maintain the stability of chloroplast thylakoid membrane, thus enhancing the photosynthetic capacity (Bajguz and Hayat, 2009; Naeem et al., 2012). Therefore, the regulation of Chl components and photosynthesis may be another strategy for GR24 to help plants adapt to LL stress.

Lipid peroxidation is used to monitor ROS damage, it reflects a basic cell membrane reactive damage under abiotic stress (Shikanai et al., 1998; Suzuki et al., 2012). ROS can react with nucleic acids, proteins and lipids, destroying cellular structure and function, and even causing cell death (Xu et al., 2010; Suzuki et al., 2012). To overcome the effects of ROS, the antioxidant system is activated (Figure 7), which is the intracellular physiological regulation in response to environmental stimuli. In addition, H_2_O_2_ was previously demonstrated to regulate abiotic stress through an ABA-dependent signaling pathway (Xia et al., 2015). SLs were exhibited to positively regulate abiotic stress response through ABA signaling, and ABA, in turn, could induce the antioxidant enzyme activity (Rhoads et al., 2006; Ha et al., 2014). The observed lower levels of MDA and H_2_O_2_ content in the group with GR24 application (Table 3), accompanied by significant increases of SOD, CAT, and POD activity in our study were reasonable to predict a potential complex crosstalk in the regulatory pathway of GR24 on tomato low light responses.

According to Ralph and Gademann (2005), only 84% of the light energy reaching leaves can be absorbed, and only 50% of the absorbed energy is distributed to photosystems. In the process of energy conversion, photosynthetic electrons are transported on the thylakoid membrane by a series of electron transport carriers (e.g., PSII, PQ, cytochrome b6f, plastocyanin, and PSI, etc.) and finally to NADP^+^ in the stroma (Genty et al., 1989; Yamori et al., 2011). Chl fluorescence signal and its measured parameters have been successfully used to probe and elucidate injury to the photosystem from various stress (Kooten and Snel, 1990; Baker, 2008). Fv/Fm and Y(II) represent the capacity of the photon energy absorbed by PSII to be used in photochemical processes under dark-adapted and light-adapted conditions, respectively (Schreiber, 2004; Shu et al., 2016). In this study, the Fv/Fm and Y(II) declined in the LL stressed tomato leaves, which implied the decrease of absorbed quanta which converted into chemically fixed energy by the increase of quanta which dissipated into heart and fluorescence (Kooten and Snel, 1990; Baker, 2008). And, the increase in both Fm and Fo (Figure 2, 3 and Table 2), indicating a blockage of electron transfer from the primary acceptor PQ (Q_A_) to the secondary acceptor PQ (Q_B_) at the acceptor side of PSII (Kooten and Snel, 1990; Baker, 2008). Application of GR24 improved the decrease of Y(II), ETR(II) and quantum efficiencies or flux ratios (e.g., φRo, φEo, φo, φPo), simultaneously mitigated the increase of Y(NO) under LL stress, indicating that GR24 might alleviate the photosynthetic processes by maintaining the stability of PSII supercomplex or enhancing the turnover of D1 protein as well as improving the photosynthetic electron transport and requirements for ATP and NADPH in the Calvin cycle (Genty et al., 1989; Turan et al., 2014; Zhou et al., 2015; Lu et al., 2017a). A high NPQ value and a low 1-qP value after GR24 treatment also indicated the samples alleviated the degree of photoinhibition by higher the capacity of heat dissipation pathway and lower the excitation pressure of PSII reaction center (Yuan et al., 2010; Shu et al., 2016). Moreover, GR24 application significantly upregulated the expression of *psbA*, *psbB*, *psbD*, *psbP*, and *cab* of LL-stressed tomato leaves (Figure 4A–F), indicating that the PSII reaction center proteins as well as assembly and functional proteins were effectively protected at the transcriptional level. Additionally, the decline of Y(I) in LL-treated leaves could be due to the increase in donor-side limitation of PSI, as reflected by Y(ND) (Figure 3), suggesting that a proportion of reduced electron carriers cannot be oxidized on PSI donor side. Exogenous GR24 treatment enhanced the size of PQ pool to facilitate PQ pool oxidization and stimulated a higher CEF to transfer electrons from PSI to PQ in LL-stressed tomato leaves (Supplementary Figures S4, S5). It demonstrated that GR24 application had a certain effect that was beneficial to absorbed light energy distribution to PSI reaction center and electron accumulation mitigation on the donor side of PSI (Terashima et al., 1994; Takahashi et al., 2009; Huang et al., 2011). In addition, the *psaA* and *psaB* expression of tomato plants under LL condition were also enhanced by application of GR24 (Figure 4G,H), indicating that GR24 could positively affect the expression of PSI-reaction-center-related proteins, to maintain PSI stability and reduce secondary damage caused by photoinhibition of PSI (Klughammer and Schreiber, 1994; Florian et al., 2009; Mayzlish-Gati et al., 2010). The values of Y(NPQ), ETR(II) and so on had a dynamic massive changes from day to day, we speculated these observations might be owing to the plant acclimatization to the growth environment. Since damage degree of PSII and PSI was alleviated, hence, the improvement of energy distribution, heat dissipation, photosynthetic electron transport chain activity and key gene expression could be the necessary regulatory pathways of GR24 on LL stress responses.

Hormonal crosstalk is spotlighted known to regulate specific phenotypes and adapt environmental stress, including heat/chilling/salinity/light stress, by regulating growth, development, source/sink transitions (Munné-Bosch and Müller, 2013). Interaction between SLs and auxin is well-demonstrated and SL biosynthesis is regulated by auxin. Exogenous auxin up-regulates *MAX3* (more auxiliary growth) and *MAX4* genes involved in SL biosynthesis (Arite et al., 2007; Hayward et al., 2009). SL mediates shoot branching through suppression of the auxin transport system by inhibiting *PIN-FPRMED* (*PIN*) activity (Prusinkiewicz et al., 2009). GR24-mediated signaling can be reversed by application of synthetic auxin 2,4-D in tomato (Koltai, 2015). The *max* mutant shows slower stomatal closure regulated by ABA and increased stomatal density, leading to decreased ABA responsiveness and increased leaf water loss rate under dehydration stress (Ha et al., 2014). It means that SL and ABA crosstalk plays an important role in integrating stress signals toward stomatal development and function. In addition, it must be noted that SLs are considered to induce LHCB genes (Mayzlish-Gati et al., 2010) and SLs and ABA are both carotenoid-derived hormones, hence there may exist a potential crosstalk between the light harvesting pathways and these two hormones. More efforts in the future could provide a better picture of these crosstalks and their interaction to better understand the SLs regulation of plant abiotic stresses.

## Conclusion

Exogenous GR24 application on tomato seedlings alleviates low light stress damage with regard to least three aspects including mitigating growth inhibition, improving photosynthetic performance and alleviating oxidative stress. In addition, application of GR24 effectively alleviates the photoinhibition of PSII and PSI under LL stress mainly by balancing excitation energy and promoting the electron transfer chain between two photosystems, thus enhancing CEF, PQ pools and quantum yield of PSII and PSI photochemistry.

## Author Contributions

WJ and HY participated in the design of the study. TL and LC conducted the experiments and collected the data. TL drafted the initial manuscript. QL contributed to the writing of the manuscript. All authors have given final approval for the publication.

## Conflict of Interest Statement

The authors declare that the research was conducted in the absence of any commercial or financial relationships that could be construed as a potential conflict of interest.
