# Supplementary material for: Improving Plant Growth and Alleviating Photosynthetic Inhibition and Oxidative Stress From Low-Light Stress With Exogenous GR24 in Tomato (Solanum lycopersicum L.) Seedlings
Source: Front Plant Sci. 2019 Apr 16;10:490. doi: 10.3389/fpls.2019.00490 (PMC6477451; doi:10.3389/fpls.2019.00490)
Supplement: Supplementary file 1 [file Table_1.DOCX]

Supplementary Material

**SUPPLEMENTARY TABLE S1 |** Gene accession numbers and primer sequences of the genes described in this study.

| Genes | Accession Numbers | Primers sequences (5’-3’) |
| --- | --- | --- |
| *psaA* | ABC56300 | F5'- GCACTAGGCCCAATGTGAGT-3'  R5'- TTTCGAGGAATGGGCCAGAC-3' |
| *psaB* | ABC56299 | F5'- TGCCAGCCTCTTTCTGGGATT-3'  R5'- ACATCCATGCCCAAACCGAT-3' |
| *psbA* | ABC56280 | F5'- GCCTGAGACACAATAGAACC - 3'  R5'- TAAGTAAGCAAGGAGGGAAC -3' |
| *psbB* | XM010321037.1 | F5’-GGGCATATATGATACCTGGGC-3’  R5’-ACAATCCAGCCTTCTCCTCC-3’ |
| *psbC* | DQ347959.1 | F5'-TTGGAGGAGAAGGGTGGATT- 3'  R5'- GCAATGAAACCAAAGACGGC-3' |
| *psbD* | AM087200.3 | F5’-GTGTATTGGGCGCTGCTTT-3’  R5’-TCTTCGGCTTGAGTTGGGTT-3’ |
| *psbP* | NM001247180.3 | F5'- CAACAGTGGGAGGAAAAGAG - 3'  R5'- GCAACTCATCTCAGCACCAT -3' |
| *cab* | M17558.1 | F5’-TCGGAGCAAACACCAT-3’  R5’-ATCCAGCCTTGAACCA-3’ |
| *Sod1* | AF034411.1 | F5'-ACCAGCACTACCAATTCTTTCT-3'  R5'-GGGGTTTAGGGGTAGTGACA-3' |
| *Cat1* | M93719.1 | F5’-GATGAGCACACTTTGGAGCA-3’  R5’-TGCCCTTCTATTGTGGTTCC-3’ |
| *Cevi16* | NM_001247041.2 | F5’-ACAGCTCCTCCGAATTCCAA-3'  R5’-GGAATCACGAGCAGCAAGAG-3' |
| *Actin* | Q96483 | F5‘-TGTCCCTATTTACGAGGGTTATGC-3'  R5‘-AGTTABAATCACGACCAGCABAGAT-3‘ |
