# Supplementary material for: Improving Plant Growth and Alleviating Photosynthetic Inhibition and Oxidative Stress From Low-Light Stress With Exogenous GR24 in Tomato (Solanum lycopersicum L.) Seedlings
Source: Front Plant Sci. 2019 Apr 16;10:490. doi: 10.3389/fpls.2019.00490 (PMC6477451; doi:10.3389/fpls.2019.00490)
Supplement: Supplementary file 2 [file Table_2.DOCX]

Supplementary Material


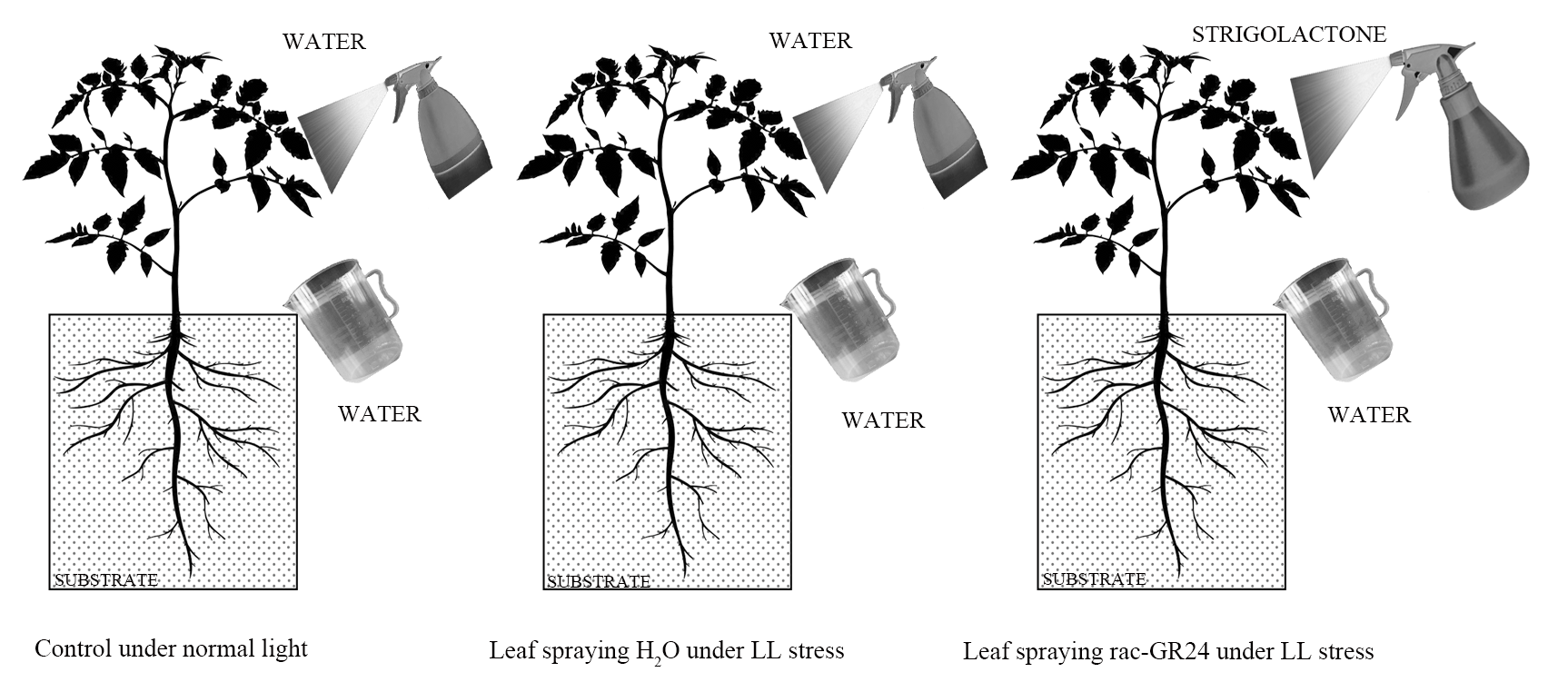


**SUPPLEMENTARY FIGURE S1** | Schematic diagram of strigolactone priming and LL-stress treatments in the experiment.

**SUPPLEMENTARY FIGURE S2** | Effects of exogenous GR24 on the plant growth of tomato seedlings under LL stress.


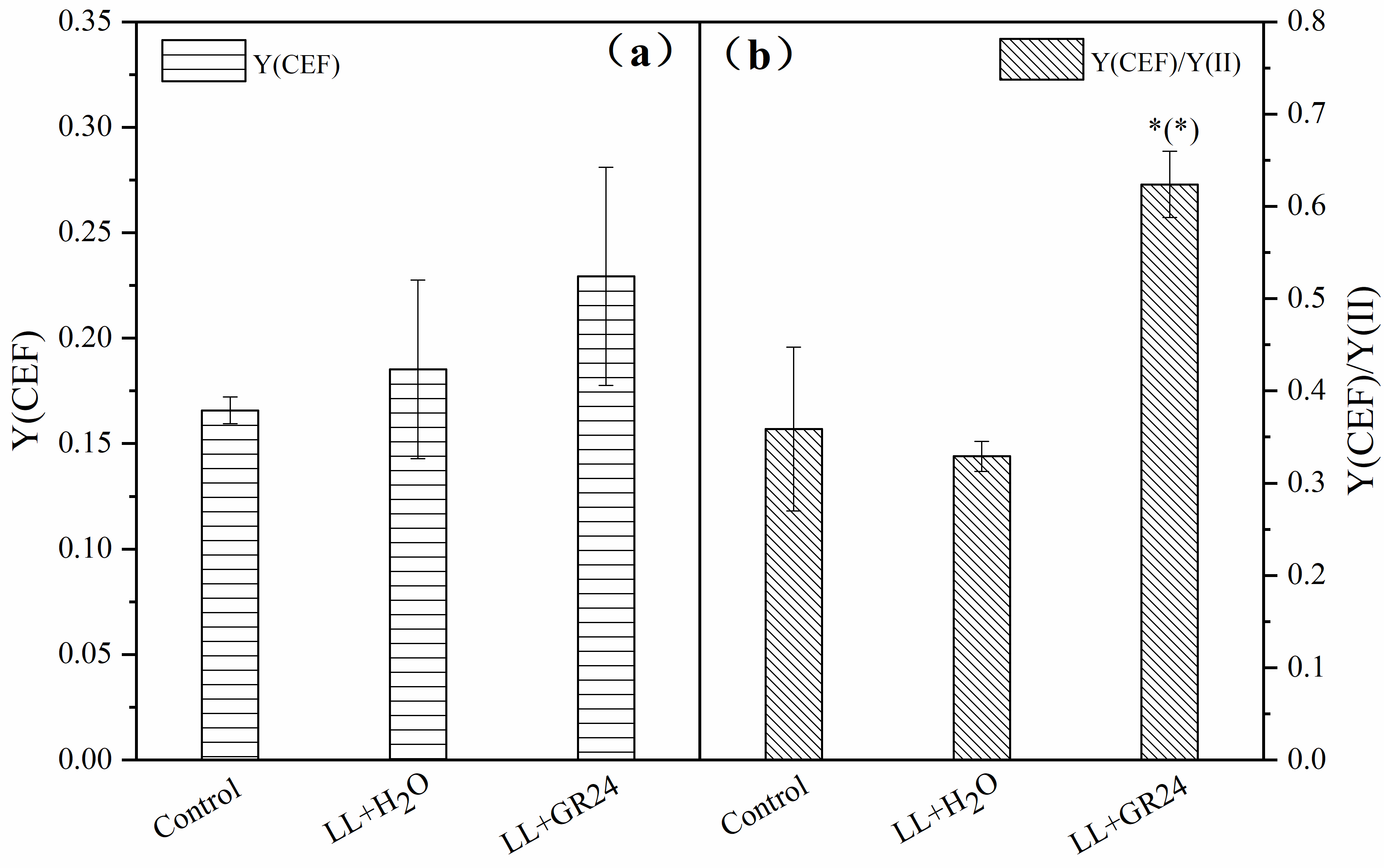


**SUPPLEMENTARY FIGURE S3** | Effects of exogenous GR24 on the cyclic electron flow in tomato leaves under LL stress. The ratio of the effective quantum yield of cyclic electron flow [Y(CEF)] to the effective quantum yield of PSⅡ [Y(Ⅱ)] was used to estimate the operation of the cyclic electron flow.


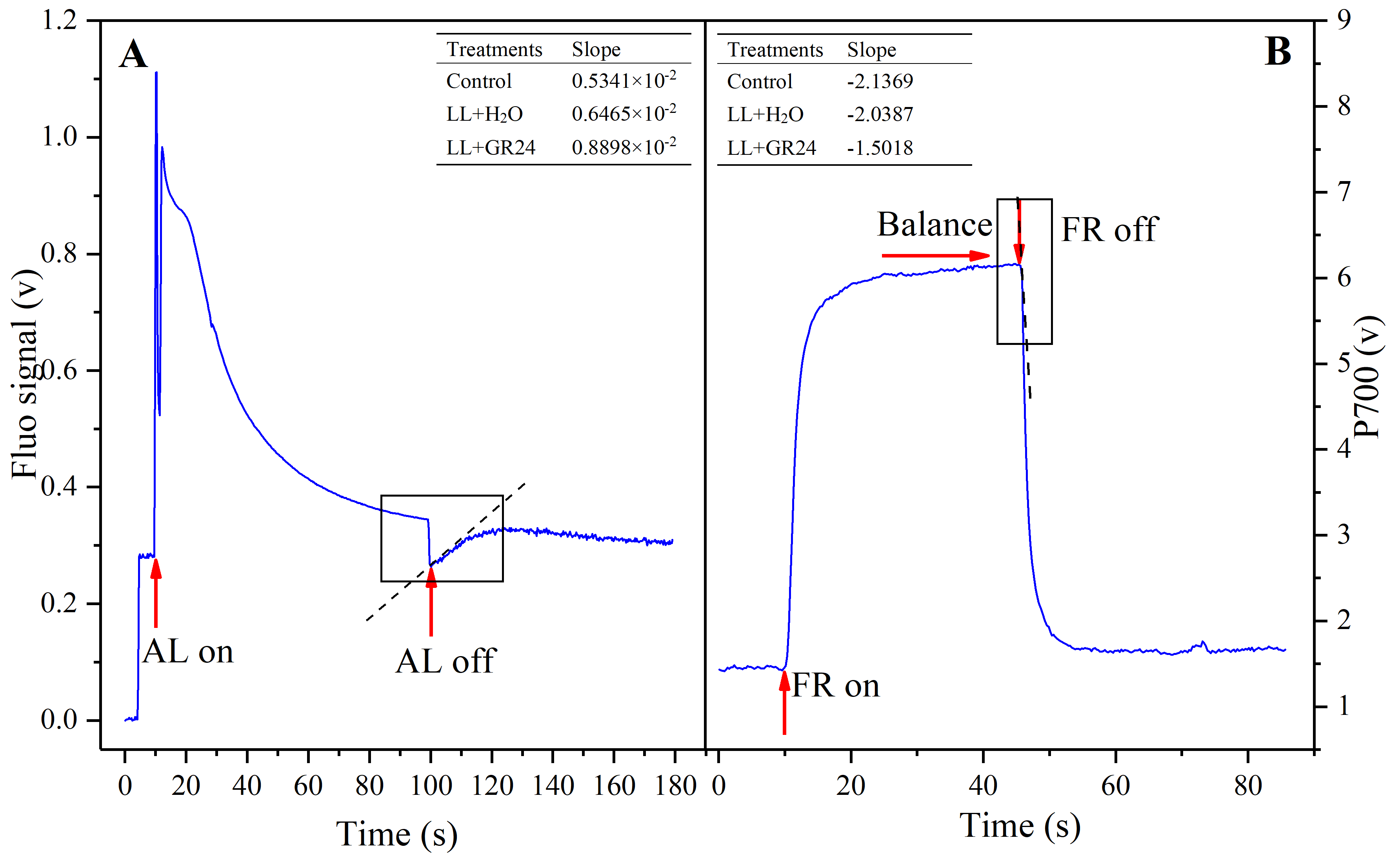


**SUPPLEMENTARY FIGURE S4** | Analysis of post-illumination chlorophyll fluorescence transient (PIFT) (a) and kinetics of the redox of P700 (b) in tomato leaf.


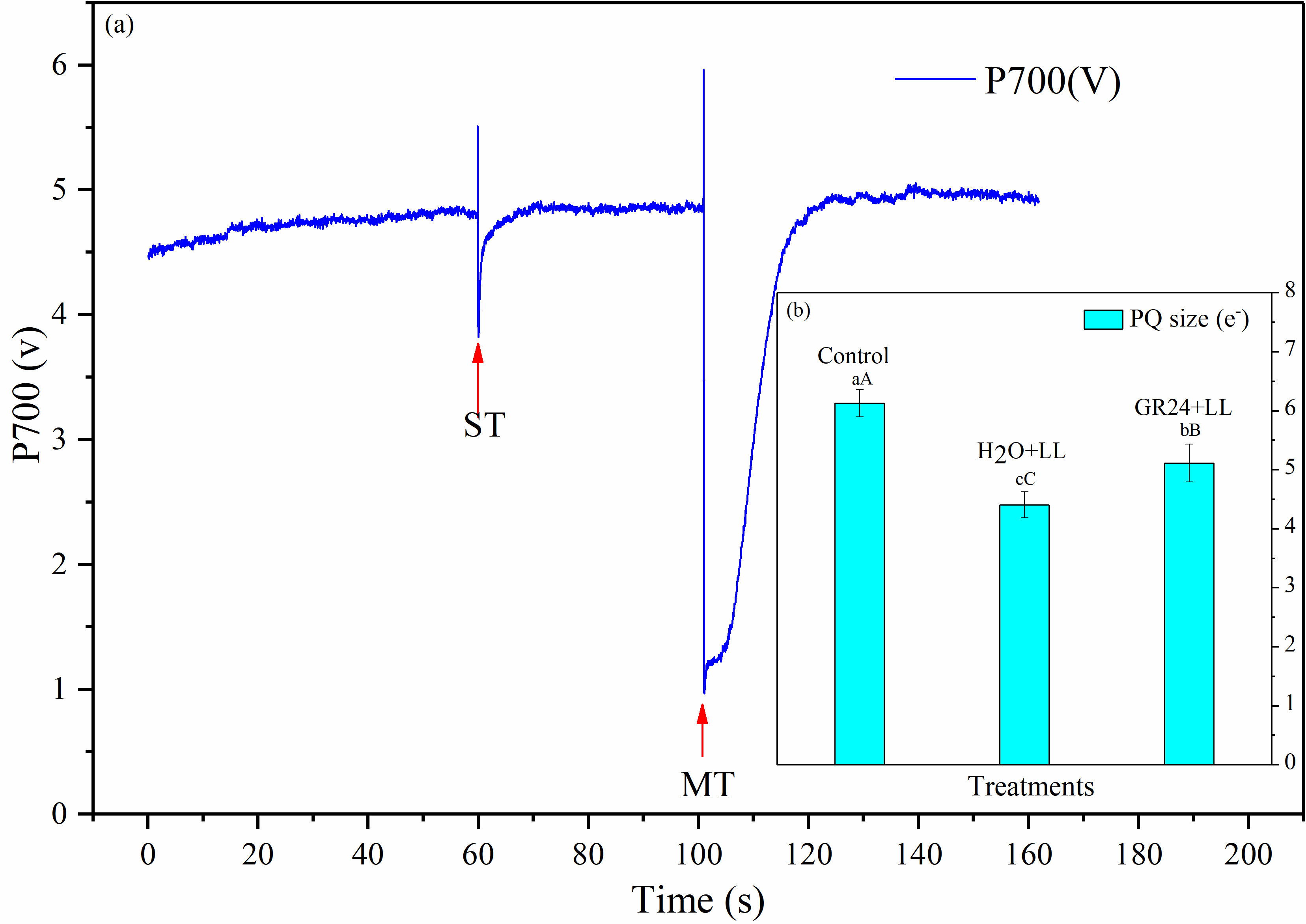


**SUPPLEMENTARY FIGURE S5** | Effects of exogenous GR24 on plastoquinone pool of tomato seedlings under LL stress.
